# Magnetic nanoparticles sensitize MCF-7 breast cancer cells to doxorubicin-induced apoptosis

**DOI:** 10.1186/1477-7819-10-62

**Published:** 2012-04-25

**Authors:** Khaled Aljarrah, Nizar M Mhaidat, M-Ali H Al-Akhras, Ahmad N Aldaher, BA Albiss, Khaled Aledealat, Fawzi M Alsheyab

**Affiliations:** 1 Biophysics laboratory, Jordan University of Science and Technology, Irbid, Jordan; 2 Magnetic measurements laboratory, Jordan University of Science and Technology, Irbid, Jordan; 3 Faculty of Pharmacy, University of Science and Technology, Irbid, Jordan

**Keywords:** Nanoparticles, MCF-7, Doxorubicin, Breast cancer

## Abstract

**Background:**

Resistance of breast cancer cells to the available chemotherapeutics is a major obstacle to successful treatment. Recent studies have shown that magnetic nanoparticles might have significant application in different medical fields including cancer treatment. The goal of this study is to verify the ability of magnetic nanoparticles to sensitize cancer cells to the clinically available chemotherapy.

**Methods:**

The role of iron oxide nanoparticles, static magnetic field, or a combination in the enhancement of the apoptotic potential of doxorubicin against the resistant breast cancer cells, MCF-7 was evaluated using the MTT assay and the propidium iodide method.

**Results:**

In the present study, results revealed that pre-incubation of MCF-7 cells with iron oxide nanoparticles before the addition of doxorubicin did not enhance doxorubicin-induced growth inhibition. Pre-incubation of MCF-7 cells with iron oxide nanoparticles followed by a static magnetic field exposure significantly (*P* < 0.05) increased doxorubicin-induced cytotoxicity. Sensitization with pre-exposure to the magnetic field was dose-dependent where the highest cytotoxicity was seen at 1 tesla. Further experiments revealed that the anti-proliferative effect of this treatment procedure is due to induction of apoptotic cell death.

**Conclusions:**

These results might point to the importance of combining magnetic nanoparticles with a static magnetic field in treatment of doxorubicin-refractory breast cancer cells.

## Background

Breast cancer is the most commonly diagnosed cancer among females world-wide. There are currently more than one million women worldwide fighting breast cancer, accounting for more than one-fifth of the global burden of cancers. In more developed and less developed countries, incidence rates vary widely, ranging from age-standardized rate 20.7 in Uganda to 90.7 in the USA per 100,000 [1]. In Europe, 421,000 cases of breast cancer among women were estimated in 2008 [2] and it is estimated that 184,450 new cases of invasive stages were diagnosed in the USA in 2008 and 230,480 in 2011 [3]. In Jordan, according to the Jordan Cancer Registry’s 2008 Cancer Incidence in Jordan Report, breast cancer constituted 35.3% of all cancer cases in women [4].

Resistance of breast cancer cells to available chemotherapeutics is a major obstacle to successful treatment. Although the cure rate from excision of the primary tumor is high, once the disease spreads to distant sites it is usually incurable by current systemic therapies, such as chemotherapy, radiotherapy, hormonal therapy and immunotherapy. The development of a novel approach for early detection, treatment and overcoming resistance of cancers is potentially required [5].

Anthracyclines are some of the most commonly used anticancer agents. The first generation of anthracyclines were isolated from the pigment-producing *Streptomyces peucetius* and were named doxorubicin and daunorubicin [6]. Doxorubicin (Adriamycin), a powerful drug in the fight against cancer, joined the oncologic practice in the late 1960s [7]. It exerts antitumor activity through its inhibition of topoisomerase II, and thus prevents chain unfolding and separation in DNA replication as well as DNA repair. This ultimately leads to cell death, called apoptosis. Other mechanisms that might be involved are intercalating into DNA, resulting in inhibition of DNA synthesis and function, and formation of cytotoxic oxygen free radicals that results in single- and double-stranded DNA breaks with subsequent inhibition of DNA synthesis and function.

Iron oxide nanoparticles have been employed in many biomedical applications due to their attractive properties, such as stability over time, biocompatibility and sensitivity to a magnetic field. Furthermore, their capability in many potential applications in biology and medicine has been explored, such as a contrast agent in magnetic resonance imaging (MRI) [8-12], a carrier in drug delivery [13-17], and as heating agents in the presence of alternating magnetic fields. This property can be very efficient in killing cancer cells through hyperthermia since these magnetic particles can be guided to the tumor by the external magnetic field gradient [18-23]. Furthermore, recent study has shown that the application of the static magnetic field in the presence of these particles can reduce cell viability, apoptosis and cell cycle aberrations [24].

In the present study, we hypothesized that magnetic nanoparticles might have anticancer activity or are able to sensitize cancer cells to the clinically available chemotherapy.

## Methods

### Cell line

Human breast cancer cell line MCF-7 was generously provided by Dr. Rick F. Thorne, from the Newcastle University, NSW, Australia, and cultured in DMEM containing 10% FCS (Bio Whittaker, Verviers, Belgium).

### Chemical reagents

Doxorubicin, purchased from Sigma-Aldrich (St. Louis, MO, USA),was dissolved in dimethyl sulfoxide (DMSO) and made up in a stock solution of 1 mM. Iron oxide nanoparticles coated by PVP (polyvinyl pyrorolidone), Propidium Iodide and 3-(4,5-dimethylthiazol-2-yl)-2,5-diphenyltetrazolium bromide (MTT) were purchased from Sigma Aldrich (Sigma-Aldrich).

### Preparation of the Fe_2_O_3_ nanoparticles

**Figure 1:**
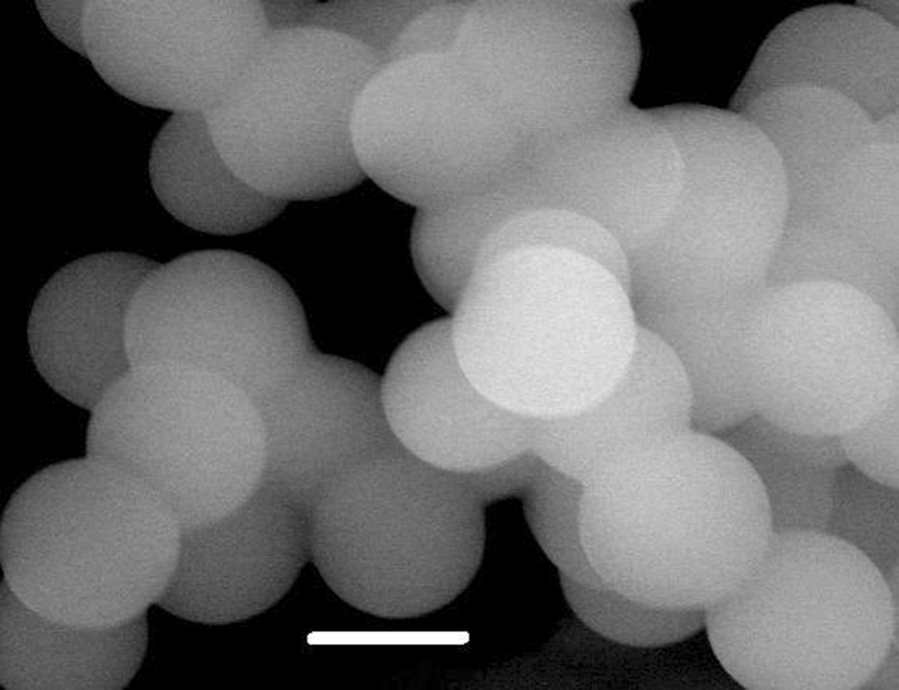
**Representative micrograph for Fe2O3 nanoparticles.** (The scale bar is 50 nm).

FeCl_3_·6H_2_O (Sigma Aldrich, >99%) and glycine (Sigma Aldrich, 99%) were mixed to create a solution of 0.1 M Fe3+, 0.2 M glycine with a total volume of 50 mL. The solution was then transferred to a Teflon-lined stainless steel vessel. The vessel was tightly sealed and heated to 150°C for 10 hours and slowly cooled to room temperature. The pH of the solution was controlled before and after the hydrothermal process. The precipitate powder was washed repeatedly with deionized water as well as absolute ethanol and sonicated for 5 minutes prior to filtering and drying in a vacuum for 10 hours. The morphology and the microstructure of the powder were characterized using X-ray diffraction (XRD) and scanning electron microscope (SEM). The average size of the spherical-shaped nanoparticles was found to be about 50 nm. A representative micrograph of the nanoparticles is shown in Figure 1 (the scale bar is 50 nm).

### Cell viability assays

The acute cytotoxic effect of doxorubicin and drug combinations on MCF-7 cells was determined using MTT assays as described previously [25]. Briefly, cells were seeded at 5,000/well onto flat-bottomed 96-well culture plates and allowed to grow for 24 hours before the desired treatment. Cells were then labeled with MTT from the Vybrant MTT Cell Proliferation Assay Kit (Molecular Probes, Eugene, OR, USA) according to the manufacturer’s instruction and the resulting formazan was solubilized with DMSO. Absorbance was read in a microplate reader at 540 nm.

### Apoptosis

Quantitation of apoptotic cells by measurement of sub-G1 DNA content using the PI method was carried out as described elsewhere [26]. In brief, MCF-7 cells were adhered overnight in a 24-well plate and incubated with different treatment combinations. Floating and adherent cells were then harvested and incubated overnight at 4°C in the dark with 750 μl of a hypotonic buffer (50 μg/mL PI in 0.1% sodium citrate plus 0.1% Triton X-100; Sigma) before flow cytometric analysis using a FACScan flow cytometer (Becton Dickinson, San Jose, CA, USA).

### Statistical analysis

Data are expressed as mean ± SE. The statistical significance of intergroup differences in normally distributed continuous variables was determined using Student’s *t*-test. *P-*values ≤ 0.05 were considered statistically significant. *P-*values ≤ 0.05 and ≤ 0.001 are indicated by * and **, respectively.

## Results and discussion

**Figure 2:**
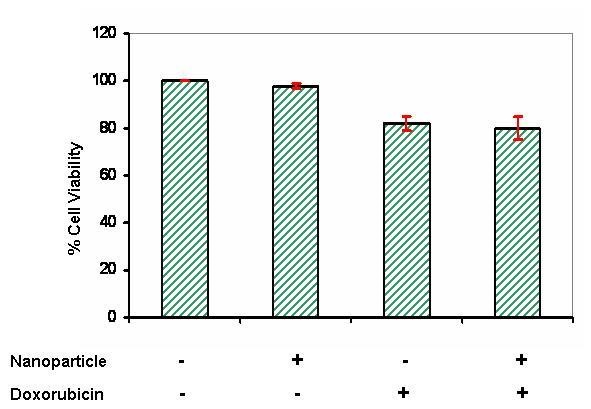
**Doxorubicin effect on growth of MCF-7 breast carcinoma cells.** Cells were incubated with doxorubicin at 1 μM or with iron oxide nanoparticles or both for 72 hours and then analyzed for cell growth using MTT assay. Control groups were treated with DMSO alone. Each value represents the mean ± SE of three independent experiments performed with quadruplicate culture.

In the present study, we evaluated the potential of spherical iron oxide nanoparticles (Fe_2_O_3_) to sensitize MCF-7 breast cancer cells to doxorubicin-induced cytotoxicity. MCF-7 cells were pre-incubated with or without Fe_2_O_3_ (at 0.5 mM) for 16 hours before the addition of doxorubicin at 1 μM for another 72 hours. As shown in Figure 2, pre-incubation with Fe_2_O_3_ did not induce cytotxic effect against MCF-7 cells and did not sensitize MCF-7 cells to doxorubicin-induced cell growth inhibition. Unlike previous reports [24], these results showed that iron oxide nanoparticles might not be the predominant source of the cytotoxicity.

**Figure 3:**
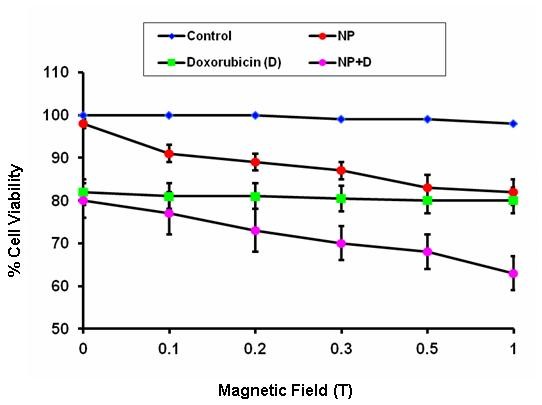
**Effect of SMF on doxorubicin-induced cell cytotoxicity.** Cells were exposed to different strengths of SMF before being incubated with doxorubicin at 1 μM or with iron oxide nanoparticles or both for 72 hours and then analyzed for cell growth using MTT assay. Control groups were treated with DMSO alone. Each value represents the mean ± SE of three independent experiments performed with quadruplicate culture.

Previous studies have shown that nanoparticles hold promise for a variety of biomedical applications due to their properties of Visualization under magnetic resonance imaging (MRI), heating with radio frequency and movement in an external magnetic field. Magnetic guidance is one of the strategies for the accumulation of nanoparticles and drugs in an affected area. To study the effect of static magnetic field (SMF) on cytotoxicity induced up on treatment with a combination of doxorubicin and Fe_2_O_3_, different strengths of SMF were used. Results in Figure 3 show that pre-exposure to SMF at different strengths did not sensitize cancer cells when treated with doxorubicin alone. Moreover, sensitization of cancer cells to doxorubicin by SMF was dose-dependent and seemed to be Fe_2_O_3_-dependent.

**Figure 4:**
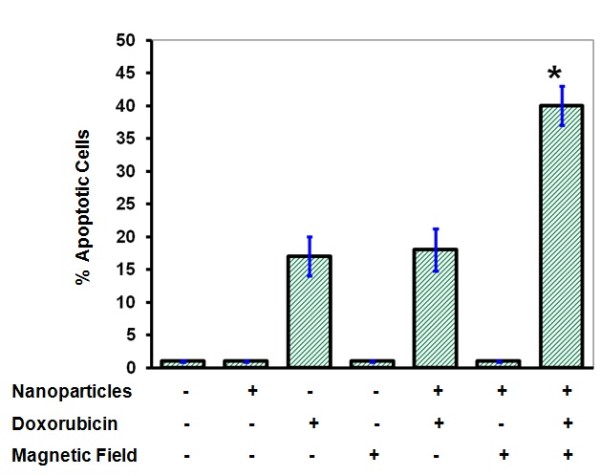
**Effect of SMF or iron oxide nanoparticles on doxorubicin-induced cell apoptosis.** Cells were exposed to SMF at 1 tesla before being incubated with doxorubicin at 1 μM or with iron oxide nanoparticles or both for 72 hours and then analyzed for cell apoptosis using the propidium iodide method. Results represent the mean ± SE of three independent experiments.

We next studied if the growth inhibition occurred by pre-exposure to SMF was due to induction of cellular death. MCF-7 cells were pre-incubated with Fe_2_O_3_ for 16 hours before they were exposed to SMF at 1 tesla and followed by the addition of doxorubicin for another 72 hours. As shown in Figure 4, cells pre-incubated with Fe_2_O_3_ and exposed to the SMF before the treatment with doxorubicin experienced a significant (*P* < 0.05) increase in the sub-G1 DNA fragmentation indicating that such treatment might sensitize cancer cells to doxorubicin-induced apoptotic cell death.

Several cellular compartments, such as lipids, carbohydrates and nucleic acids, might be damaged by increased levels of reactive oxygen species (ROS) in that increased levels of ROS might induce irreversible damage to DNA [27,28]. Cell death inducers, such as chemotherapy and ionizing radiotherapy, have been shown to induce their cytotoxicity through ROS generation [29-32]. Our results showed that treatment with Fe_2_O_3_ particles alone are unable to inhibit cancer cell growth largely due to the inability to generate ROS (data not shown). Other studies showed that in the presence of a SMF, FeCl_2_ induces DNA damage in an ROS-dependent manner indicating that SMF, somehow, has a role in activating ROS generation from FeCl_2_[33-35]. This might be not the situation in the present study since FeCl_2_ has been removed by nanoparticle washing. Consistent with our studies, it has been shown that SMF induces apoptotic cell death, particularly in human transformed cells and in rat tendon fibroblasts cell cultures [36-39]. Furthermore, results in the present study revealed that the SMF without the addition of the ROS generator, Fe_2_O_3_, or the ROS inducer, doxorubicin, is unable to induce cancer cell growth inhibition. This was consistent with previous studies showing that SMF did not enhance the rate of apoptotic cells without the addition of hydrogen peroxide [40].

The apoptotic potential of SMF might be due to the ability of SMF to induce marked alteration in cellular shape [41]. In addition to the oxidative stress induced by Fe_2_O_3_, this effect might lead to physical stress that will modulate the intracellular levels of calcium. Alteration in the intracellular levels of calcium has been shown to induce a response called unfolded protein response (UPR). The later occurs due to increased accumulation of mis- or malfolded proteins. Persistence of UPR signaling in presence of doxorubicin and Fe_2_O_3_, the cell will die by induction of the endoplasmic reticulum (ER) stress [42].

Another proposed mechanism for cell death is that SMF might inhibit DNA repair procedures [43,44]. Thus DNA damage induced by topoisomerase I inhibitor, doxorubicin, will not be repaired and cells will commit suicide, probably by induction of one or more of the ER stress signaling arms [42].

## Conclusions

Collectively, these results indicate that intra-tumor implantation of Fe_2_O_3_ followed by exposure to a SMF and doxorubicin might provide a promising approach in the treatment of patients with breast cancer and for those being refractory to doxorubicin. This is strongly supported by previous findings, indicating that iron oxide particles have long blood retention rates and are biodegradable [5].

## Abbreviations

DMSO = Dimethyl sulfoxide; DMEM = Dulbecco’s modified Eagle’s medium; ER = Endoplamic reticulum; FCS = FACScan flow cytometer; MRI = Magnetic resonance imaging; PVP = Polyvinyl pyrorolidone; ROS = Reactive oxygen species; SEM = Scanning electron microscope; SE = Standard Error; SMF = Static magnetic field; UPR = Unfolded protein response; XRD = X-ray diffraction.

## Competing interests

The authors declare that they have no competing interests.

## Authors’ contributions

All authors substantially contributed to the manuscript and approved the final submission.
